# Quantifying Spillover of an Urban Invasive Vector of Plant Disease: Asian Citrus Psyllid (*Diaphorina citri*) in California Citrus

**DOI:** 10.3389/finsc.2022.783285

**Published:** 2022-02-16

**Authors:** Brett R. Bayles, Shyam M. Thomas, Gregory S. Simmons, Matthew P. Daugherty

**Affiliations:** ^1^Department of Global Public Health, Dominican University of California, San Rafael, CA, United States; ^2^Department of Natural Sciences and Mathematics, Dominican University of California, San Rafael, CA, United States; ^3^Department of Fisheries, Wildlife and Conservation Biology, University of Minnesota, St. Paul, MN, United States; ^4^United States Department of Agriculture, Animal and Plant Health Inspection Service, Salinas, CA, United States; ^5^Department of Entomology, University of California, Riverside, Riverside, CA, United States

**Keywords:** biological invasion, invasion dynamics, landscape ecology, urban-rural interface, herbivore spillover, citrus greening disease, huanglongbing

## Abstract

Urban environments frequently play an important role in the initial stages of biological invasions, often serving as gateways for non-native species, which may propagate to nearby natural and agricultural ecosystems in the event of spillover. In California, citrus trees are a dominant ornamental and food plant in urban and peri-urban environments. We studied the invasion dynamics of the Asian citrus psyllid (*Diaphorina citri*), which became widespread in urban areas of southern California starting in 2008, to understand the factors driving its more recent invasion in commercial citrus groves. Using a multi-year monitoring database, we applied a suite of models to evaluate the rate at which groves accrued their first *D. citri* detection and the cumulative number of detections thereafter. Grove characteristics and landscape context proved to be important, with generally higher invasion rates and more cumulative detections in groves that were larger, had more edge, or had more perforated shapes, with greater urbanization intensity favoring more rapid invasion, but with inconsistent effects of distance to roads among models. Notably, distance to urban or other grove occurrences proved to be among the most important variables. During the early phase of *D. citri* invasion in the region, groves closer to urban occurrences were invaded more rapidly, whereas more recently, invasion rate depended primarily on proximity to grove occurrences. Yet, proximity to urban and grove occurrences contributed positively to cumulative *D. citri* detections, suggesting a continued influx from both sources. These results suggest that inherent features of agroecosystems and spatial coupling with urban ecosystems can be important, temporally dynamic, drivers of biological invasions. Further consideration of these issues may guide the development of strategic responses to *D. citri*'s ongoing invasion.

## Introduction

Urban ecosystems provide a suite of services for human health and well-being, including food production from urban gardens and commercial agriculture (1–3). However, such benefits should be weighed against potential disservices created as a result of these human-altered landscapes (4, 5). In particular, urban areas contain unique sets of social and ecological processes that may create focal points for the introduction, establishment and spread of non-native species (6–10).

Anthropogenic landscapes often exist along gradients of different land-use types, including urban and peri-urban cities, commercial agriculture, and a range of natural ecosystems (8, 11, 12). The particular spatial configurations of these landscapes in a given area are idiosyncratic, which suggests that a diverse interplay of social and ecological processes determine the initial risk of invasion, as well as if and when spillover into surrounding urban forest landscapes will occur (4, 10, 13, 14).

Cross-habitat spillover involving the movement of invasive species between agroecosystems and natural habitats have been documented (15, 16). Most studies have focused on asymmetric movement of insects between agricultural areas and surrounding natural habitats (13, 15, 17). Landscape features, such as the extent and characteristics of habitat edges and the relative proximity to source habitats, have been shown to affect spillover across habitats (13). Such spillover events can further influence community dynamics of the recipient habitat through processes such as parasitism, predation, and disease transmission (13, 14, 18, 19).

Southern California, a region characterized by extensive urban development, has recently experienced the introduction of numerous phytophagous insects (20). Of the ~10 exotic arthropod introduction events per year in the state, an estimated 20% become invasive pests (21). One invader of particular concern is the Asian citrus psyllid (*Diaphorina citri*) because of its ability to transmit the bacterium *Candidatus* Liberibacter asiaticus (*C*Las) associated with huanglongbing (HLB; citrus greening disease) [Grafton-Cardwell et al., (22)]. Symptoms of HLB, to which all commercial varieties of citrus are considered susceptible, include progressive yellowing of leaves, stunting of shoots, defoliation, reduced yield and fruit quality, and tree mortality (23). Citrus growing regions where both the pathogen and vector successfully invaded, such as Florida, have faced severe economic impacts (24).

**Figure 1:**
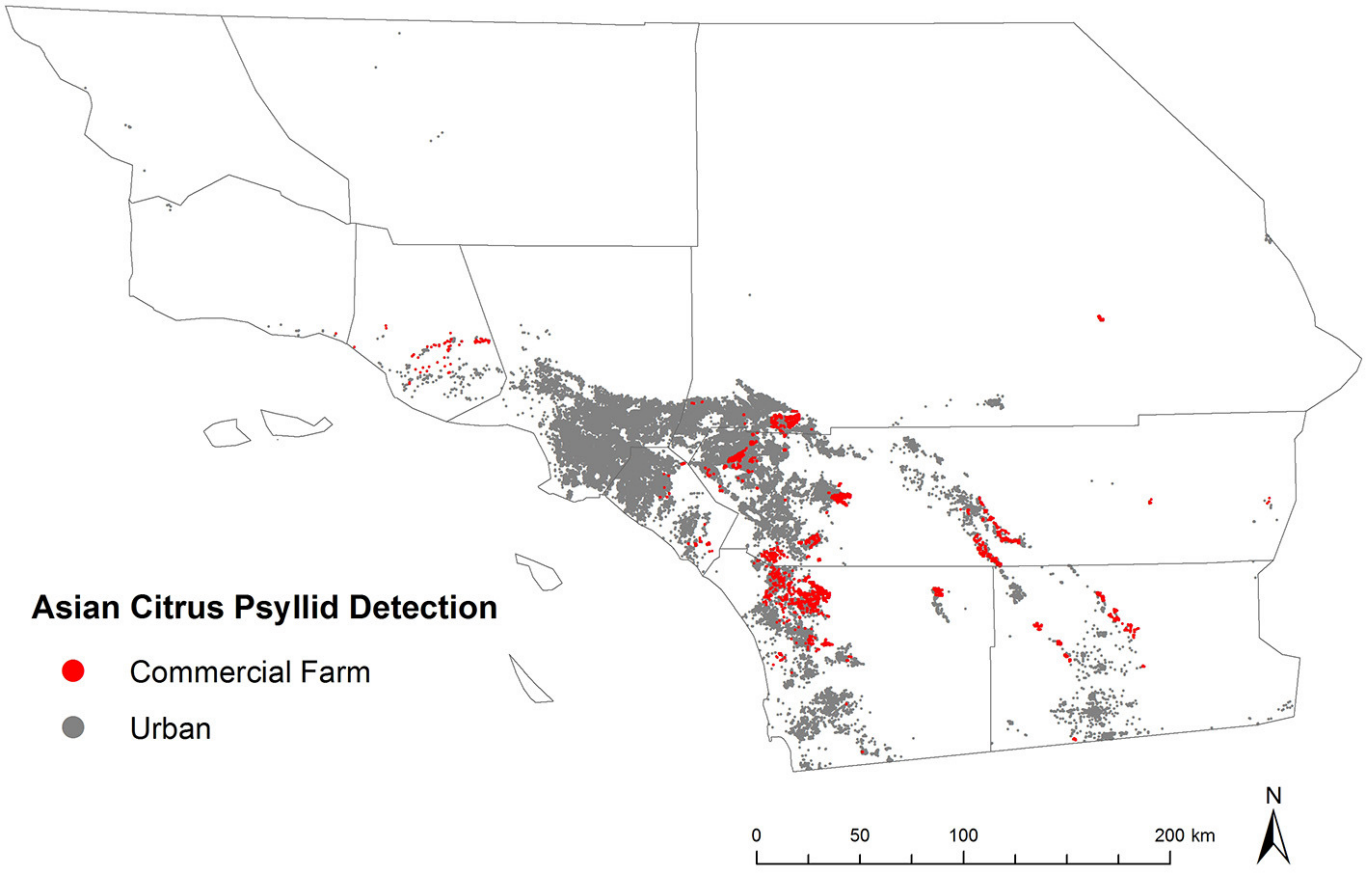
Map of Southern California showing the distribution of commercial citrus groves and surrounding urban land cover.

Citrus has unique historical and cultural significance in California, following its likely introduction into the region by Spanish missionaries in the late 18th century (25). The first commercial production began in southern California in the mid-19th century, expanded to other areas of the state in subsequent decades, and eventually developed into what is currently estimated to be approximately 270,000 ac of citrus farmed at a yearly production value of more than $3B (25–27). The development of the citrus industry is credited with contributing to California's population expansion throughout the 19th century, the creation or evaluation of novel citrus varieties that are commonly grown today, and the widespread acceptance of biological control as an element of integrated pest management (25, 28). Enthusiasm for citrus carries over to urban and other residential areas of California where it is frequently grown as an ornamental and food plant. Surveys conducted in select neighborhoods in southern California estimated that more than 60% of properties had at least one citrus tree present (29). Thus, residential and commercial trees are both at risk to invasive pests and diseases, such as *D. citri* and huanglongbing, and indeed may exacerbate damage to each other in the event of cross-habitat spillover (Figure 1).

*D. citri* was first detected in California in a residential area of San Diego County in 2008 and in Los Angeles by 2009 (30). In response, a strategic plan was implemented to mitigate the impact of this invasive pathosystem in California (27). Over the next few years, the insect spread throughout urban and peri-urban areas, apparently driven by favorable climatic conditions and landscape characteristics in these environments (30, 31). *D. citri* was first detected in a handful of commercial citrus groves in 2011, and over the next few years became increasingly prevalent in these settings (30). Finally, the first case of HLB was detected in a residential area of Los Angeles County in 2012 (32). Disease surveys have subsequently identified upward of 2500 infected trees that have been removed to limit pathogen spread (27, 33). Thus far, all cases of the disease have occurred in residential areas, but there was one detection of a *C*Las positive psyllid from a commercial grove, and nearby groves are considered at risk.

While invasive species spillover between agricultural and natural ecosystems has been quantified, less is known about how common spillover is from urban to agricultural ecosystems, or the processes governing it across space and time. The general sequence of *D. citri* occurrence and patterns of *C*Las spread in Southern California are consistent with urban environments playing a potentially important role in facilitating the establishment of *D. citri* (30, 31, 33). Yet, formal analyses are lacking that identify the factors driving *D. citri* invasion of commercial citrus groves, which is critically important for refining disease detection and management strategies. Here, we quantify the magnitude of urban-to-agriculture *D. citri* spillover, and the underlying role of grove-level characteristics, urban landscape-context, and neighborhood proximity in governing the spatial and temporal distribution of *D. citri* activity in commercial groves.

## Materials and Methods

### Data

We obtained a georeferenced database of 78,000 *D. citri* panel trap collection sites in Southern California from the California Department of Food and Agriculture (CDFA) and the Citrus Pest and Disease Prevention Program (CPDPP) via the Citrus Research Board (CRB) from 2008 through 2014. Detection records were obtained from 14 x 23 cm double-sided yellow panel traps that were positioned in parts of 10 counties (Imperial, Kern, Los Angeles, Orange, Riverside, Santa Barbara, San Bernardino, San Diego, and San Luis Obispo) [see (30) for a detailed description of the trapping database].

We developed three indicators of “grove-level” characteristics, including: (1) grove area, (2) grove edge, and (3) grove perforation. Grove area was calculated as the size of each commercial citrus grove in square meters using a commercial citrus grove (definition from 26) layer (obtained from the CRB) in ArcGIS v. 10.2. Given that the number of traps varied with the size of the grove, we expect to find an area effect in the analyses, and therefore use it in part to minimize confounding effects on the estimation of other variables in the models.

Because there is a biologically plausible mechanism by which an “edge effect” in citrus groves can impact insect abundance (34) and disease transmission risk (23), we used the Landscape Fragmentation Tool (LFT v2.0) to quantify grove edge and grove perforation (35). Grove edge was calculated as the proportion of citrus tree pixels within 100 meters of the citrus grove/non-citrus grove outside edge. Grove perforation was calculated as the proportion of citrus tree pixels along the edge of small clearings found within groves. Together, these landscape metrics characterize the extent to which citrus groves are fragmented and are therefore an effective proxy for quantifying the edge effect (36).

We developed two indicators of “urban landscape-level” characteristics surrounding groves, including: (1) urbanization intensity, and (2) proximity to transportation corridors (i.e., distance to roads). Urbanization was calculated as the proportion of both treeless cover and impervious surface within 100 m of each grove:


$$\begin{array}{l} \frac{\;\left[\left(100\%\;-\;\%\;Tree\;Cover\right)\;+\;\%\;Impervious\;Surface\right]}{2} \end{array}$$

This produced an index of urbanization intensity ranging from 0 (low urbanization) to 100 (high urbanization). Proximity to transportation corridors was calculated as the minimum distance between groves and the nearest road designated for commercial truck traffic by the California Department of Transportation (available from http://www.dot.ca.gov/hq/tsip/gis/datalibrary/).

Finally, we developed a set of indicators to measure the “neighbor effect” to more mechanistically capture spatial linkages stemming from urban influences. We included measures of distance to urban or peri-urban *D. citri* detections to capture any spillover from urban environments, and distance to *D. citri* detections in other citrus groves to capture grove-to-grove spillover. Due to strong year-to-year collinearity among distances to urban *D. citri* detections and among distances to *D. citri* grove detections, we grouped neighbor effect indicators into two temporal phases relative to when *D. citri* detections in urban areas and in groves started to become common (30). Our indicators of neighbor effect included: (1) early-phase (2008–2011) distance between citrus groves and nearest residential citrus detection (i.e., “urban early-phase”), (2) late-phase (2012–2014) distance between citrus groves and nearest residential citrus detection (i.e., “urban late-phase”), (3) early-phase (2011–2012) distance between commercial citrus groves and nearest commercial grove detection (i.e., “grove early-phase”), and (4) late-phase (2013–2014) distance between commercial citrus groves and nearest commercial grove detection (i.e., “grove late-phase”). An initial principal components analysis supported grouping the neighbor effects in this manner (MP Daugherty, *unpublished data*).

### Data Analysis

We used two sets of analyses to identify the factors related to *D. citri:* (1) the invasion rate of commercial citrus groves (i.e., why some groves were invaded more quickly than others), and (2) the cumulative number of *D. citri* detections in all invaded groves as a proxy for the potential impact of this invasive vector (i.e., why some groves have more repeat detections than others). Cumulative detections were calculated as the total number of traps in a grove, to date, that collected at least one *D. citri* (i.e., total number of traps with *D. citri* present). We adopted this approach, instead of using explicit *D. citri* counts on each trap, due to concerns about inconsistency in reporting counts versus simply reporting *D. citri* presence absence. Prior to analysis, all grove, landscape, and neighbor effects were standardized by converting them to z-scores. This standardization is important to ensure that the different units in which the variables were measured (e.g., grove area [m^2^] vs. urbanization [0–100%]) did not bias the model fitting and selection process in favor of particular variables (37). All analyses were conducted using the R programming language (v. 3.6.1).

For the analysis of invasion rate, we developed a pair of Cox proportional hazard models, which are semi-parametric tests often used to test “time-to-event” data that includes multiple variables (38), to discern the effects of grove-level variables in an urban landscape-level context from that of neighbor effects that capture spillover pressure [(39); coxph() in R]. The first model evaluated the effects of grove-level characteristics (i.e., grove area, grove edge, grove perforation) and urban landscape-level (i.e., urbanization intensity, distance to roads) variables on the rate at which commercial groves incurred their first *D. citri* detection. Because the distance to roads variable did not meet the proportional hazards assumption, an interaction with time was included in the model (40). Next, to capture more mechanistically the effects of potential spatially dependent drivers of invasion rate, a second model evaluated effects of grove-level characteristics and neighbor effects [i.e., grove early-phase (2011–2012); urban early-phase (2008–2011); grove late-phase (2013–2014); urban late-phase (2012–2014)] on invasion rate. Due to collinearity with some of the neighbor effects, the urban landscape-level effects were not included in the second model. For both models, we fit different formulations with all possible combinations of the 5 or 7 unique variables. The different model formulations were then ranked via Akaike Information Criterion [AIC; (41)] to identify one or more best-supported models, which we define as falling within five AIC units (i.e. ΔAIC < 5) of the highest ranked model (i.e. lowest AIC value). Finally, we used model averaging with shrinkage [model.avg() in package MuMLn; Bartoń, (42)] of the best-supported models to estimate the effect size and 95% confidence interval for each variable represented in at least one of the best-supported models. In the event that a single model substantially outperformed the others, we evaluated the significance of individual terms with a series of likelihood-ratio tests (38).

Unlike invasion rate, cumulative number of *D. citri* detections in a citrus grove is a proxy measure of vector impact, stemming from effects of vector density or frequency relative to hosts on disease outbreaks (43). We used a similar approach to analyze the effects of grove-level characteristics, urban landscape-level context, and neighbor effects on the cumulative number of grove detections (i.e. number of *D. citri* positive traps) for the approximately 500 groves that had at least one detection. Due the count nature of cumulative detections, and high frequency of groves with a low number of observed detections, they were analyzed with a pair of generalized linear models with negative binomial error (38). As before, one set of models analyzed grove-level characteristics and urban landscape-level context variables and a second analyzed grove-level characteristics and neighbor effects. In addition, each set of models included an effect of time since infestation (i.e., number of months since the first *D. citri* detection in that grove) to control for some groves being first invaded much (i.e., up to 3 years) earlier than others. Again, we fit all possible combinations of variables within each set of models, ranked those models using an information-theoretic approach, and used model averaging to estimate the effect size of coefficients in the best-supported models.

## Results

Of the nearly 2,500 unique citrus groves for which *D. citri* trapping data was available, 539 had at least one detection by the end of 2014. The earliest commercial citrus detection occurred in April 2011, with six additional groves having detections in that year, an additional 208 groves in 2012, and the remainder in 2013 and 2014. Of those groves in which *D. citri* was detected, 6.5% (35/538) had a single trap detection, but with a maximum of 359 cumulative detections (mean ± SD: 18.76 ± 33.46).

### Invasion Rate

**Figure 2:**
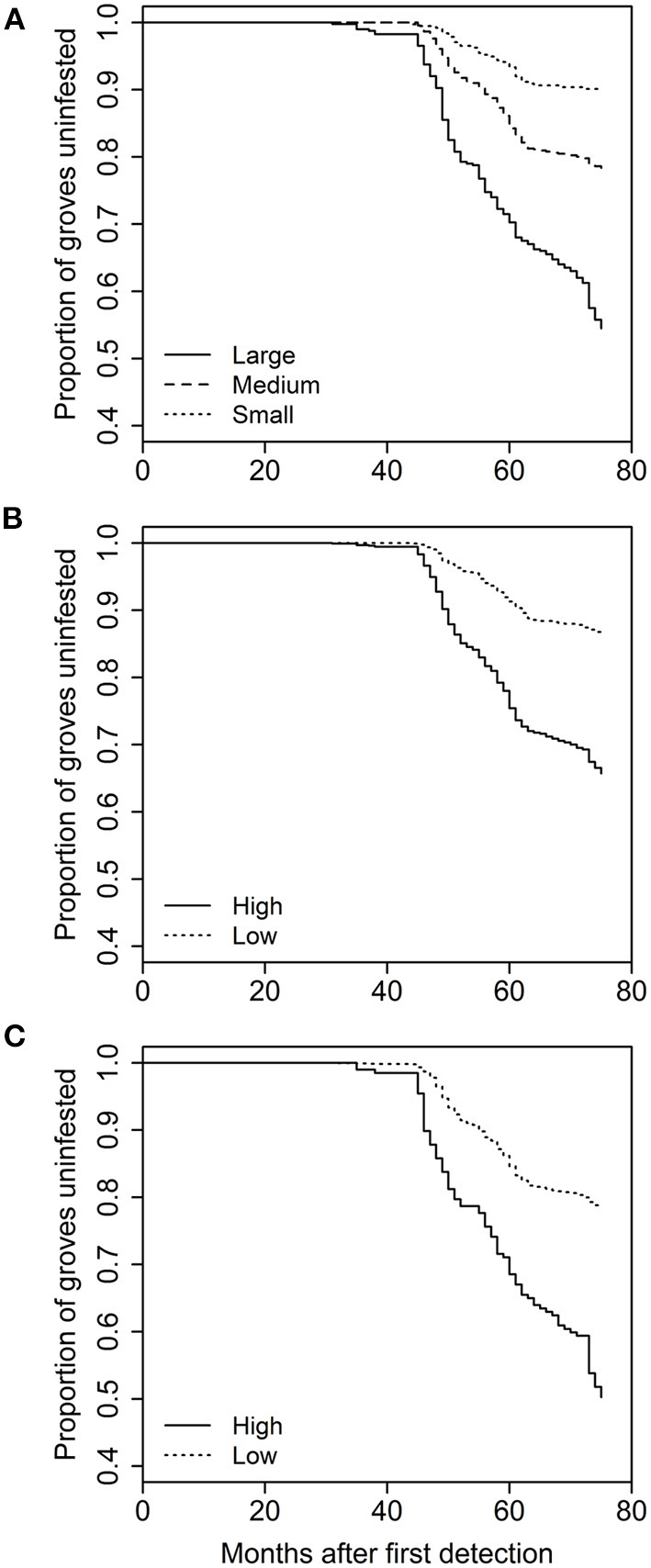
Effect of **(A)** grove area, **(B)** amount of grove edge, or **(C)** grove perforation on the time to first *D. citri* detection in a grove. Kaplan-Meir plots were created by grouping based on natural breaks in the independent variables, resulting in three relative grove sizes and two relative values for edge and perforation. The x-axis reflects the amount of time since the first detection of *D. citri* in California in 2008.

**Figure 3:**
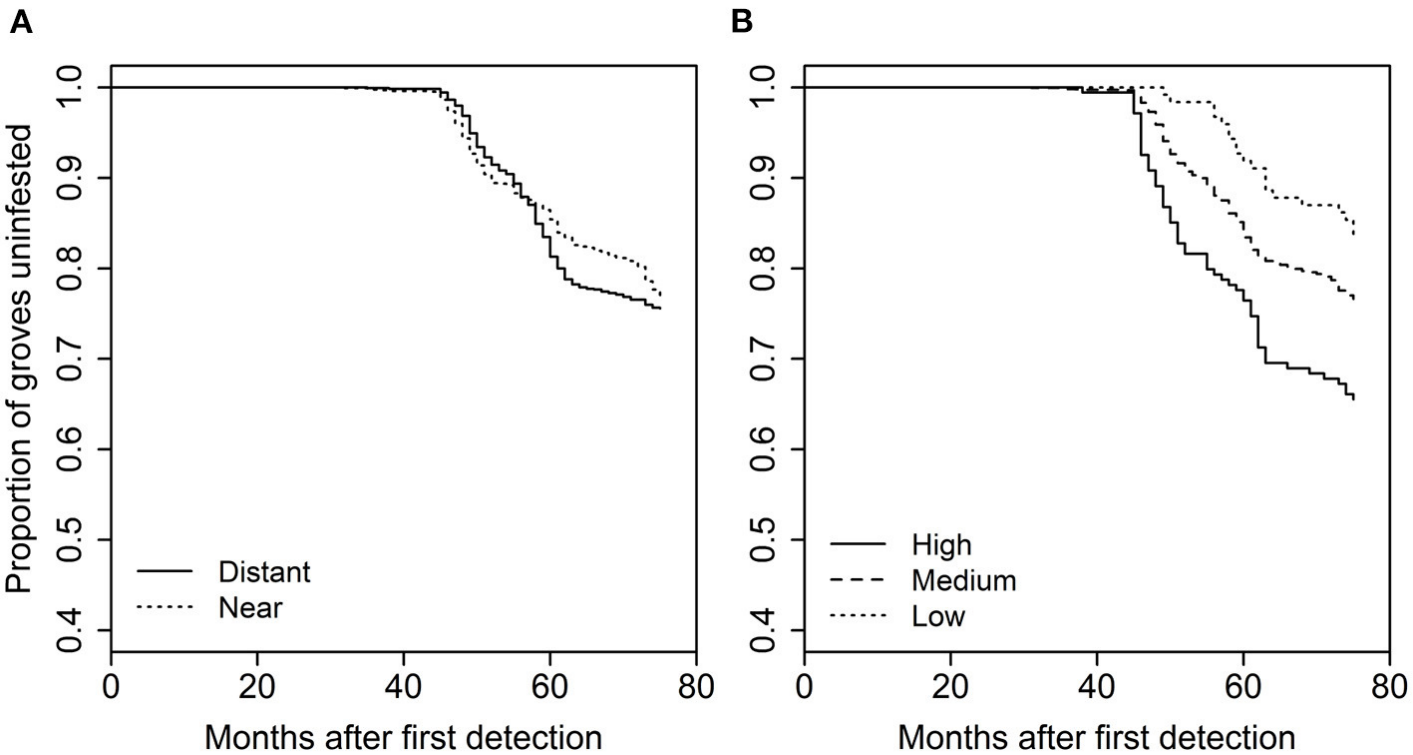
Effect of **(A)** distance to major roads, or **(B)** surrounding urban intensity on the time to first *D. citri* detection in a grove. Groupings for plots were created based on natural breaks in the independent variables, resulting in two relative values for roads and three for urban intensity.

The analysis of grove characteristics and landscape context on rate of invasion revealed the full model with all five variables and an interaction between distance to roads and time provided the best description of the data (> 5 AIC units lower than all other models). As a result, we did not use model averaging for this part of the analysis. The full model included significant effects of grove area (χ^2^ = 5.637, *df* = 1, *p* = 0.0176), grove edge (χ^2^ = 70.664, *df* = 1, *p* < 0.0001), grove perforation (χ^2^ = 23.423, *df* = 1, *p* < 0.0001), distance to roads (χ^2^ = 13.9622, *df* = 1, *p* = 0.0002), and urbanization intensity (χ^2^ = 17.672, *df* = 1, *p* < 0.0001), and a road distance-by-time interaction (χ^2^ = 19.558, *df* = 1, *p* < 0.0001). With respect to the grove-level characteristics, grove area [hazard ratio (SE) = 1.063 (0.017)], amount of grove edge [1.391 (0.04)], and the extent of grove perforation [1.15 (0.026)] were all positively related to the rate at which individual citrus groves incurred their first *D. citri* detection (i.e., hazard ratio > 1; Figure 2). Both of the urban landscape-context variables were also positively related to invasion rate [urbanization intensity: 1.197 (0.042); distance to roads: 2.167 (0.188)]. However, the road-by-time interaction with a hazard ratio <one [0.965 (0.003)] indicates that early on groves further from roadways were invaded at equivalent or lower rates than groves distant from roadways (Figure 3).

**Figure 4:**
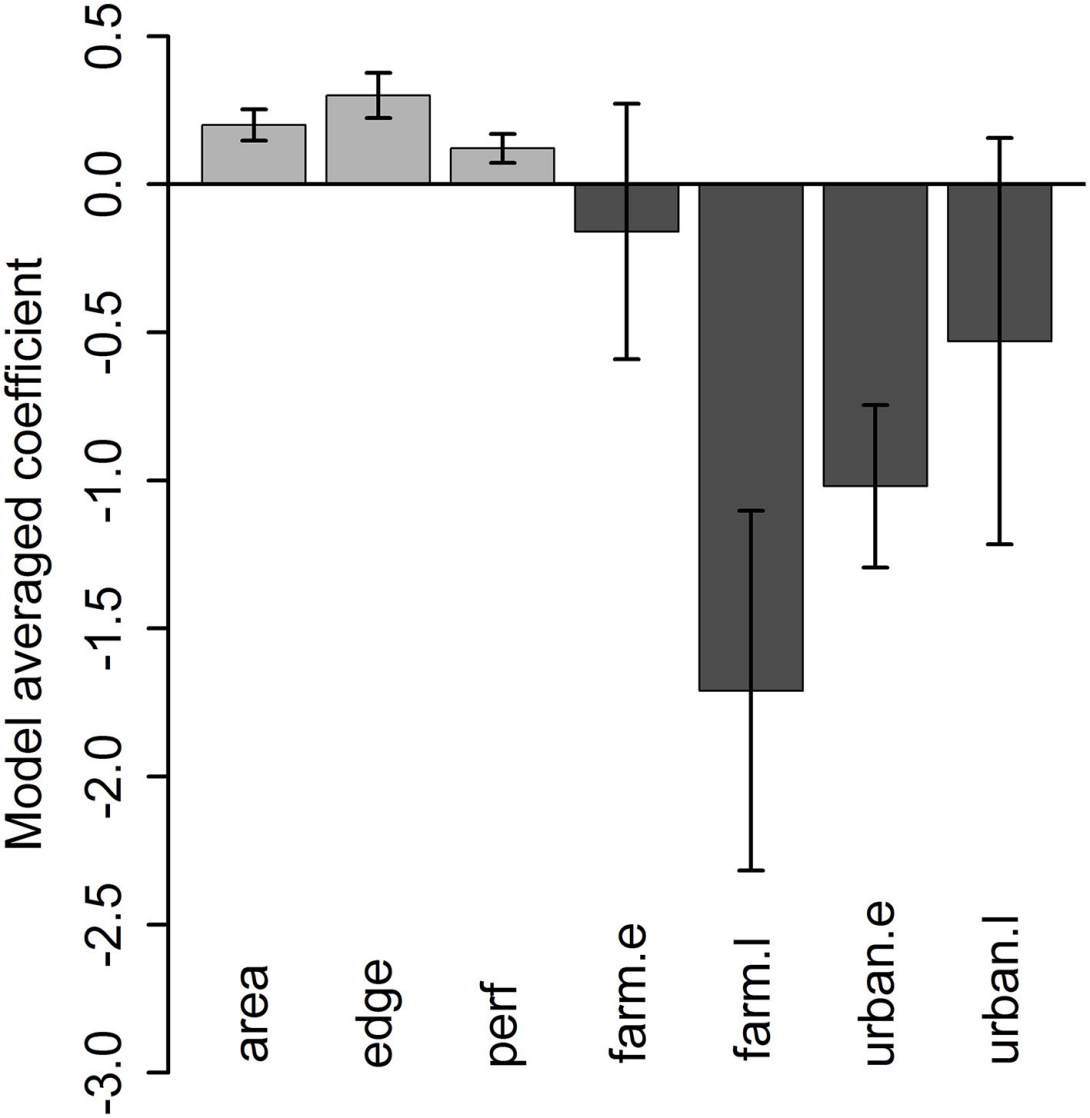
Model-averaged coefficient values (±95% confidence intervals) across the four best models for grove characteristics (grove area [“area”], grove edge [“edge”], grove perforation [“perf”]) and neighbor effects (grove early-phase distance [“grove.e”], grove late-phase [“grove.l”], urban early-phase [urban.e], or urban late-phase [“urban.l”] occurrence of *D. citri*) on time to *D. citri* first detection in a given citrus grove. Variables with error bars overlapping 0 are appropriately viewed as having minimal effects on invasion rate.

**Figure 5:**
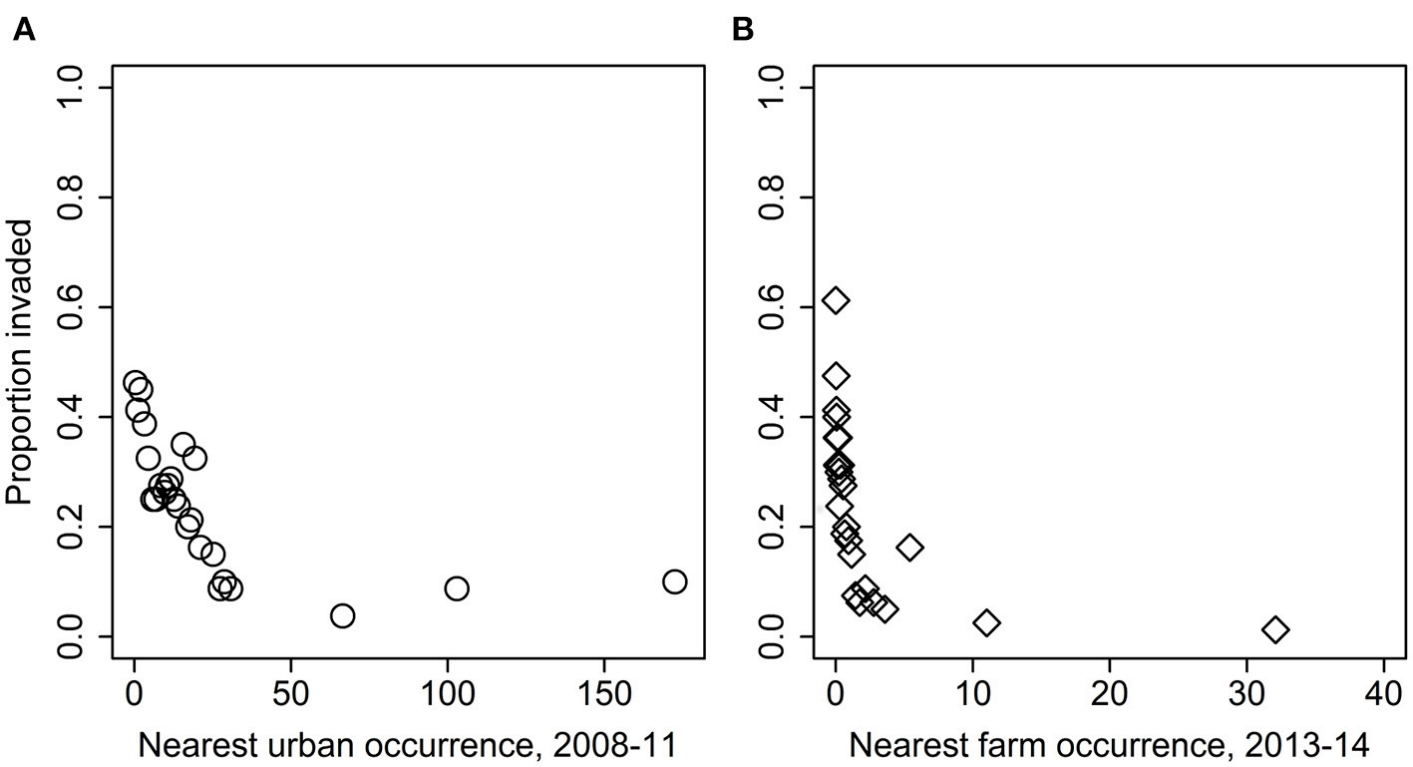
Overall proportion of groves with at least one *D. citri* detection as a function of distance (km) from the nearest **(A)** urban early-phase, or **(B)** grove late-phase occurrence of *D. citri*.

For the analysis of grove-level characteristics and neighbor proximity effects on invasion rate, of the dozens of models evaluated, four performed similarly well, with all other model formulations having AIC values at least 15 units greater (Supplementary Table S1). Based on the model-averaged coefficients for these preferred models, grove area, grove edge, and grove perforation were significantly positively related to invasion rate (Figure 4). The model-averaged coefficients indicated that both early-phase distance to nearest grove occurrence (i.e., grove early-phase) and late-phase distance to nearest urban occurrence (i.e., urban late-phase) were not significantly related to invasion rate. However, the late-phase distance to nearest grove occurrence (i.e., grove late-phase) and early-phase distance to nearest urban occurrence (i.e., urban early-phase) are strongly negatively related to invasion rate (Figure 4; Supplementary Figure S1). With respect to these last effects, the overall pattern of new *D. citri* grove detections suggest that potential spillover (i.e., the invasion kernel) from urban early-phase occurrences is strongest within 25 km and spillover from grove late-phase occurrences is strongest within 10 km (Figure 5).

### Cumulative *D. citri* Detections

**Figure 6:**
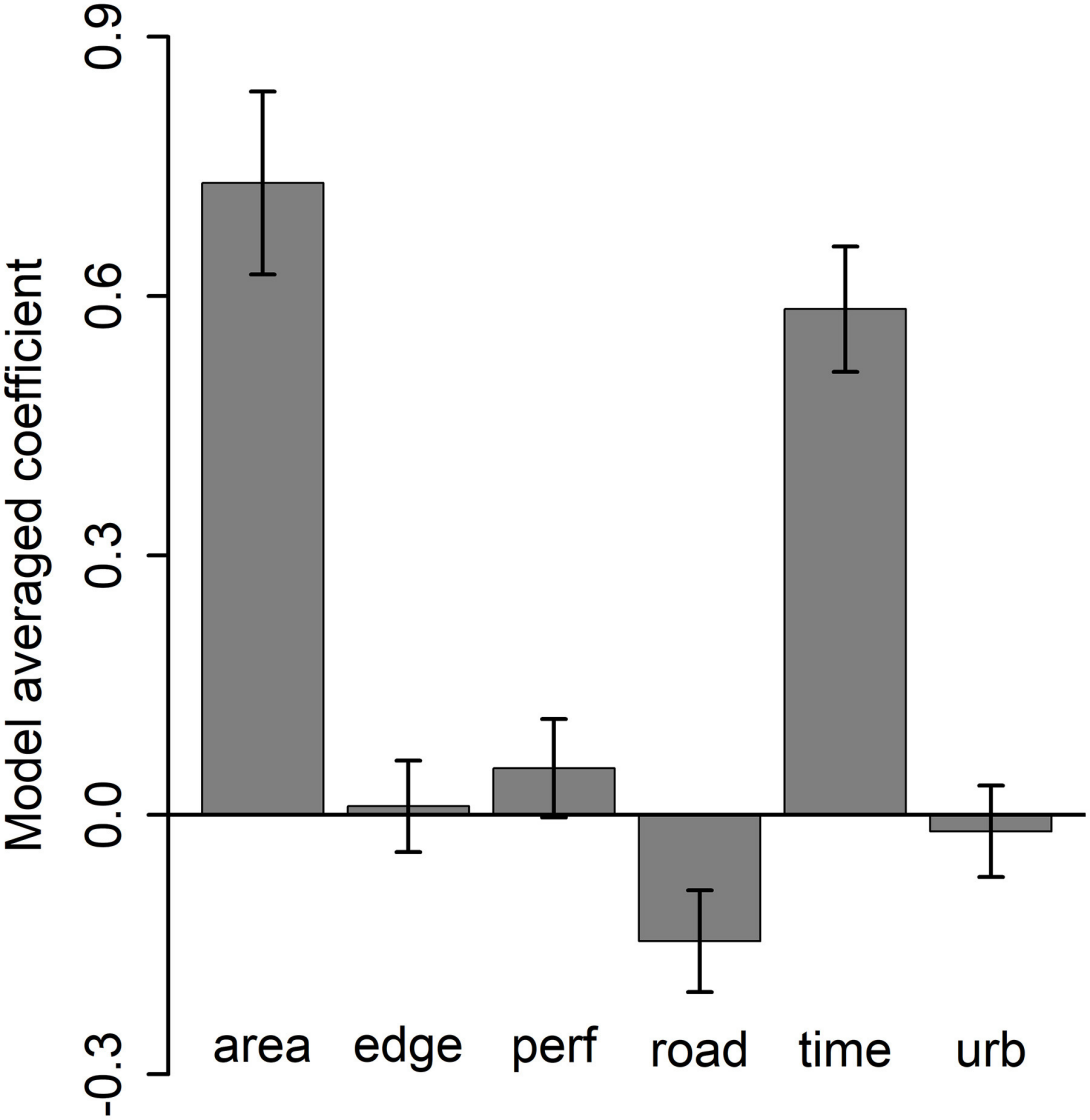
Model-averaged coefficient values (± 95% confidence intervals) across the seven best models for grove characteristics, landscape context (distance to roads [“road”], urbanization intensity [“urb”]), and temporal effects (number of months after first *D. citri* detection in the grove) on cumulative number of *D. citri* detections.

For the analysis of grove-level and urban landscape-level effects, seven models performed similarly well with respect to AIC rankings (Supplementary Table S2). Based on the model-averaged coefficients grove area and time since first detection were strongly and positively related to more *D. citri* detections (Supplementary Figure S2), distance to roads was slightly negatively related to the number of detections (Supplementary Figure S2C), and the other three variables had equivocal effects (Figure 6).

**Figure 7:**
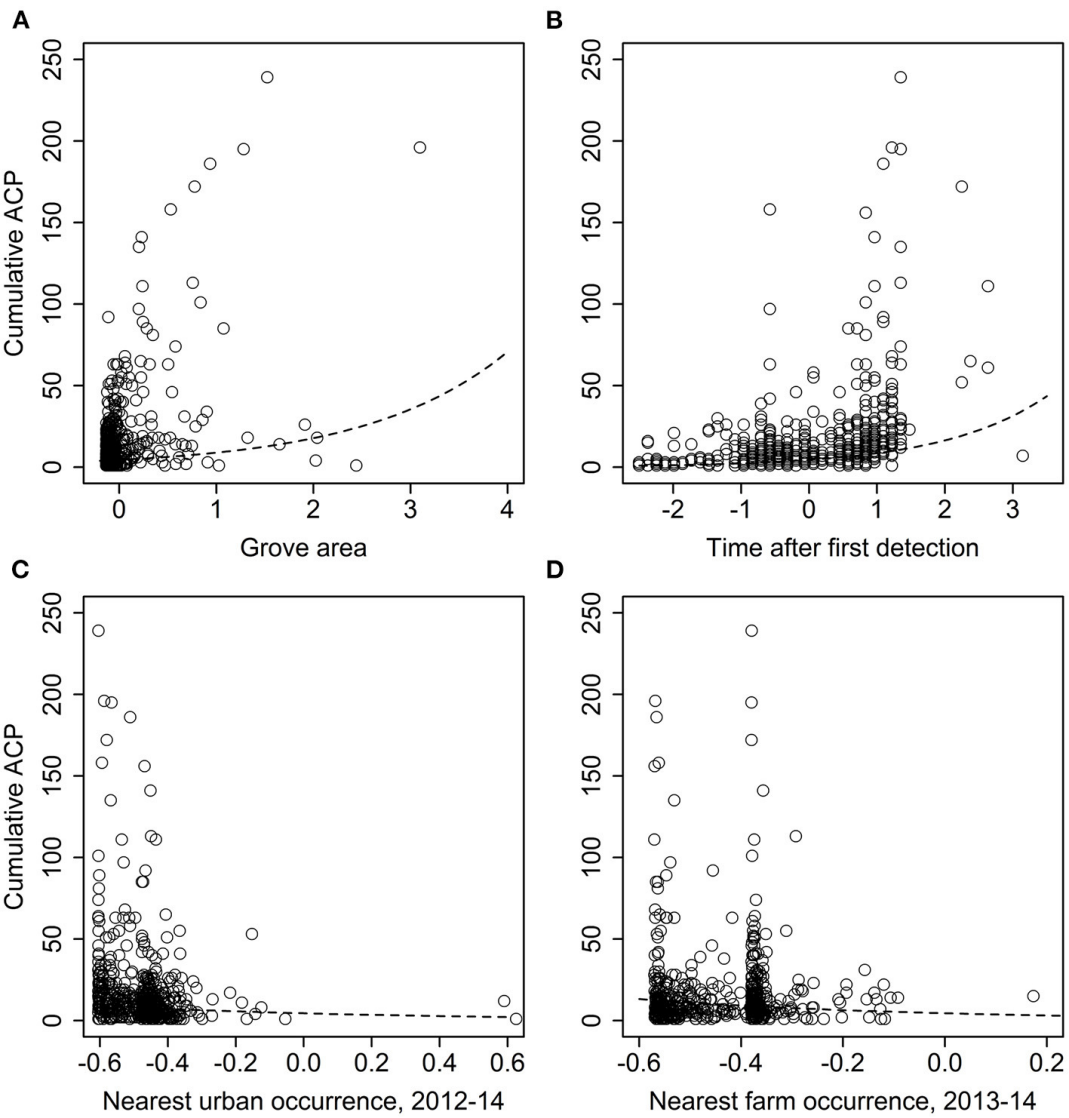
Effect of **(A)** grove area, **(B)** time since first *D. citri* detection, and distance to the nearest **(C)** grove late-phase *D. citri* occurrence or **(D)** urban late-phase occurrence, on the cumulative number of *D. citri* detections in a citrus grove. Independent variables were all standardized via z-scores; smaller or more negative values equate to smaller groves, more recent first detections, or shorter distances.

For the analysis of grove-level characteristics and neighbor effects, of the dozens of models evaluated, one substantially outperformed the others (>10 AIC units lower). That model included significant effects of grove area (χ^2^ = 55.991, *df* = 1, *p* < 0.0001), time since first detection (χ^2^ = 255.63, *df* = 1, *p* < 0.0001), urban late-phase distance (χ^2^ = 23.784, *df* = 1, *p* < 0.0001), and grove late-phase distance (χ^2^ = 24.470, *df* = 1, *p* < 0.0001). Both grove area (slope ± SE = 0.677 ± 0.09) and time since first detection (0.634 ± 0.04) were positively associated with *D. citri* detections (Figures 7A,B). Conversely, greater urban late-phase distances (−1.136 ± 0.233) and nearest grove detections (−1.737 ± 0.351) were associated with fewer cumulative *D. citri* detections (Figures 7C,D).

## Discussion

Cross-habitat spillover of organisms is increasingly gaining recognition, particularly in agroecosystems and urban forests. Many studies have focused on the movement of organisms between agricultural habitats and natural habitats (13, 15, 17). Unlike these studies, our research addresses the less familiar spillover between urban and agricultural habitats within an anthropogenic-dominated landscape, and its importance for the spread and impact of an invasive insect pest of high economic significance. In our study area, citrus constitutes a substantial fraction of urban tree plantings (29). *D. citri* was first recorded in these urban micro-habitats (such as house backyards and gardens) of southern California in 2008. Within three years, *D. citri* had invaded commercial citrus groves (30). Overall, our results highlight the role of grove-level factors in combination with two spatially-explicit factors, namely urban landscape-level context and neighbor effects in modulating urban-agricultural spillover.

At the grove-level, grove area stood out as a common factor affecting both rate of invasion and magnitude of *D. citri* infestation such that large groves were invaded faster and also contained greater cumulative detections of *D. citri*. These results are not surprising given that, as noted earlier, trap number varied grove size. Yet, part of the observed effect may also be attributable to larger plantings of citrus trees acting as larger targets for *D. citri* invasion. A similar ‘magnetic effect’ has been reported among mass flowering crops wherein pollinators tend to spillover in large numbers during blooming periods (13, 44). After controlling for grove area, the proportion of citrus along grove edges and the extent of grove perforation only affected the rate of invasion. In both cases, a higher proportion of grove edge and grove perforation increased the rate of invasion. Studies have similarly shown that the amount and ecological nature of the edge, and consequent edge-effects, are often important drivers of spillover from groves to natural habitats (15). Citrus trees at the outer edge of groves have been shown to contain a higher abundance of *D. citri* compared to trees in the interior, particularly those groves on the exterior edge of large farms (34). Interestingly, grove edge and grove perforation had no significant effect on the cumulative number of *D. citri* detections in a citrus grove, perhaps indicating that once a grove is invaded local recruitment is an important element of *D. citri* population dynamics. Studies have shown that resource availability and abundance can affect population growth and eventual abundance of organisms that spillover into high quality recipient habitats such as mass flowering crops plants (45, 46).

Both urban landscape-level variables, distance to major roads and urbanization intensity, had significant effects on the rate of *D. citri* invasion. The distance to roads had an overall negative effect on the rate of spillover during early stages of invasion, but then shifted toward a positive effect in more recent years. Urbanization intensity increased the rate of invasion, implying that citrus groves surrounded by higher levels of urbanization are more quickly invaded by *D. citri*. Taken together, the positive effect of urbanization intensity on rate of invasion suggests heavily disturbed urban-grove edges, along with the distance to roads, can facilitate introduction pathways and hasten *D. citri* invasion. Distance to major roads tends to result in greater *D. citri* abundance within groves, which again suggests the role of roads in serving as introduction pathways and potentially affecting multiple introductions of *D. citri* individuals from the surrounding landscape. An earlier study on the initial *D. citri* detections within urban Los Angeles identified major roads entering the city as a likely introduction pathway (31). In the only other study known to us, proximity to anthropogenic edges such as roads was found to enhance parasitic attacks of a leaf miner in suburban woodland fragments (15, 47). Our findings highlight the importance of incorporating urban landscape-level factors in spillover studies.

Studies have shown that cross-habitat spillover can influence community dynamics of the recipient habitat through processes such as predation, parasitism, and disease transmission (13, 14, 18, 19). While grove-level characteristics set the risk threshold for invasion, neighbor effects suggest that proximity to *D. citri* invaded neighborhoods are the eventual deterministic factor driving the spillover. In this respect, it is important to note that the early-phase distance to nearest urban occurrence (urban early-phase) and late-phase distance to nearest grove occurrence (grove late-phase) are negatively related to the rate of invasion. In other words, there appear to be strong spatial linkages between urban occurrences of *D. citri* and its arrival in nearby groves, and among groves once one of them is invaded. The relatively sporadic presence of citrus host plants in urban micro-habitats can initiate the urban-grove spillover, which suggests the need for more spatially comprehensive management plans that take into consideration the potential role of surrounding landscape.

The strong effects of early-phase urban and late-phase grove invasion implies that the spillover pressure from *D. citri* invasion is most pronounced after spreading substantially in the surrounding landscape. In a recent spillover study, the magnitude of bee spillover from mass flowering citrus groves to nearby woodland patches was similarly found to vary in both space and time, such that spillover was not only higher in woodland patches in landscapes with high cover of mass flowering citrus groves but was particularly higher after the citrus blooming period (44). The study further demonstrated that the observed spillover dynamics are largely driven by variation in flower cover across the landscape, and hence is considered to be resource-mediated. Our findings point to a similar resource-mediated mechanism behind the spillover but with the striking difference that the movement of *D. citri* individuals were initially from relatively resource-poor urban conditions to resource-rich groves. Overall, the neighbor effects suggest that spillover of *D. citri* is not merely a function of spatial proximity to nearest invaded grove and/or urban location but is also shaped by the temporal trajectory of *D. citri* invasion.

To mitigate the impact of the *D. citri* invasion in Southern California, a strategic plan was implemented that included monitoring for *D. citri* and *C*Las, removal of infected trees, the release of biological control agents, a residential insecticide treatment program, restrictions on the movement of plant material, and community outreach (27, 48). Despite this, urban-to-agriculture spillover occurred within the first few years of the southern California invasion. This observed early-phase effect of urban invasion suggests *D. citri* spillover into citrus groves was initially driven by *D. citri* from citrus plantings in urban and peri-urban locations in close proximity to groves. Later citrus grove invasions were driven largely by spillover from nearby invaded groves, which suggests that the source of *D. citri* spillover into citrus groves shifts over time. This apparent shift in spillover dynamics has implications for management of *D. citri* and similar insect pests that are not restricted to a single habitat type. Specifically, for areas where the invasion is at an advanced stage, such as in Southern California, attempts to mitigate further *D. citri* spread into commercial citrus will likely benefit more from management of invaded groves than from measures focused in residential areas (27, 48). Conversely, in areas where *D. citri* is not as well-established, such as the Central Valley of California (49), given competing demands on resources, our results indicate mitigating future *D. citri* invasion of groves is likely to benefit most from management in urban areas. Meanwhile, attempts to mitigate further spread of *C*Las and huanglongbing may require different strategies. Given that to date all of the ~1,500 documented cases of huanglongbing in California have occurred in residential citrus (33), and within cumulative counts of *D. citri* in groves indicating a continuing influx from urban areas, concentrating vector control and inoculum reduction programs (27) in urban areas is likely to be more impactful than in commercial citrus. In other words, development of appropriate management strategies requires a recognition of the fluid nature of invasion dynamics and careful consideration of the specific management objectives.

## Data Availability Statement

The raw data supporting the conclusions of this article will be made available by the authors, without undue reservation.

## Author Contributions

BB and MD: conceptualization and study design. BB, MD, and ST: statistical analysis and writing. MD: funding acquisition and supervision. GS: data acquisition. All authors contributed to the article and approved the submitted version.

## Funding

This project was supported by funding from USDA-APHIS-PPQ to MD.

## Conflict of Interest

The authors declare that the research was conducted in the absence of any commercial or financial relationships that could be construed as a potential conflict of interest.

## Publisher's Note

All claims expressed in this article are solely those of the authors and do not necessarily represent those of their affiliated organizations, or those of the publisher, the editors and the reviewers. Any product that may be evaluated in this article, or claim that may be made by its manufacturer, is not guaranteed or endorsed by the publisher.
