# Supplementary material for: Quantifying Spillover of an Urban Invasive Vector of Plant Disease: Asian Citrus Psyllid (Diaphorina citri) in California Citrus
Source: Front Insect Sci. 2022 Feb 16;2:783285. doi: 10.3389/finsc.2022.783285 (PMC10926442; doi:10.3389/finsc.2022.783285)

**SUPPLEMENTAL MATERIAL**

**Table S1.** Four best models from a survival analysis of the effects of grove-level characteristics (grove area, relative amount of grove edge [“edge”], grove perforation [“perf”]), neighbor effects (distance to nearest early-phase grove occurrence 2011-12 [“grove.early”], late-phase grove occurrence 2013-14 [“grove.late”], early-phase urban occurrence 2008-11[”urb.early”], late-phase urban occurrence 2012-14 [“urb.late”]) on the time to first *D. citri* detection in a grove.

| **Model^1^** | **AIC** | **ΔAIC** |
| --- | --- | --- |
| **area** + **edge** + **grove.late** + **perf** + **urb.early** + **urb.late** | 8714.88 | 0 |
| **area** + **edge** + grove.early + **grove.late** + **perf** + **urb.early** + urb.late | 8715.26 | 0.38 |
| **area** + **edge** + grove.early + **grove.late** + **perf** + **urb.early** | 8716.64 | 1.76 |
| **area** + **edge** + **grove.late** + **perf** + **urb.early** | 8719.30 | 4.42 |

**Table S2**. Seven best models from a multiple regression of the effects of grove-level characteristics (grove area, grove edge, grove perforation [“perf”]), urban landscape-level context (distance to major roads [“road”], urbanization intensity [“urb”]), and the time since the first occurrence of *D. citri* (time) on cumulative *D. citri* abundance.

| **Model^1^** | **AIC** | **ΔAIC** |
| --- | --- | --- |
| **area** + **perf** + **road** + **time** | 3895.3 | 0 |
| **area** + **perf** + **road** + **time** + urb | 3895.8 | 0.5 |
| **area** + edge + **perf** + **road** + **time** | 3896.8 | 1.5 |
| **area** + **road** + **time** | 3897.2 | 1.9 |
| **area** + edge + **perf** + **road** + **time** + urb | 3899.1 | 3.8 |
| **area** + **road** + **time** + urb | 3899.8 | 4.5 |
| **area** + edge + **road** + **time** | 3900.3 | 5.0 |

^1^Bolded variables are significantly associated with cumulative *D. citri* abundance

**Figure S1.** Effect of distance to nearest A) urban *D. citri* occurrence, 2008-11, or B) other commercial citrus *D. citri* occurrence, 2012-14, on the time to first *D. citri* detection. Groupings for plots were based on natural breaks in the two distance measures.


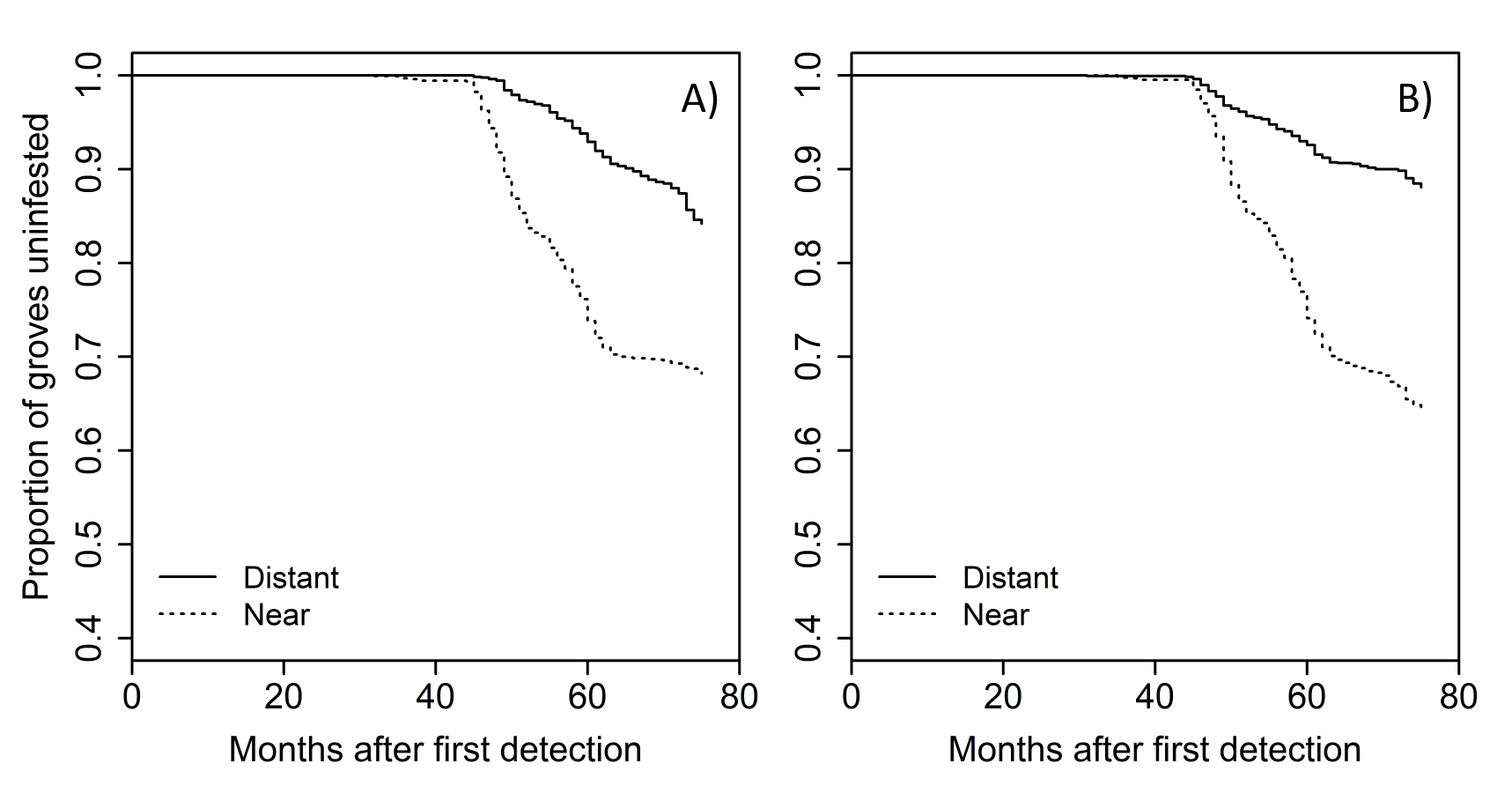


**Figure S2**. Effect of A) grove area, B) distance to roads, or C) duration of time since first *D. citri* occurrence on the cumulative *D. citri* abundance on groves. Independent variables were all standardized via z-scores; smaller or more negative values equate to smaller groves, shorter distances, or more recent occurrences.


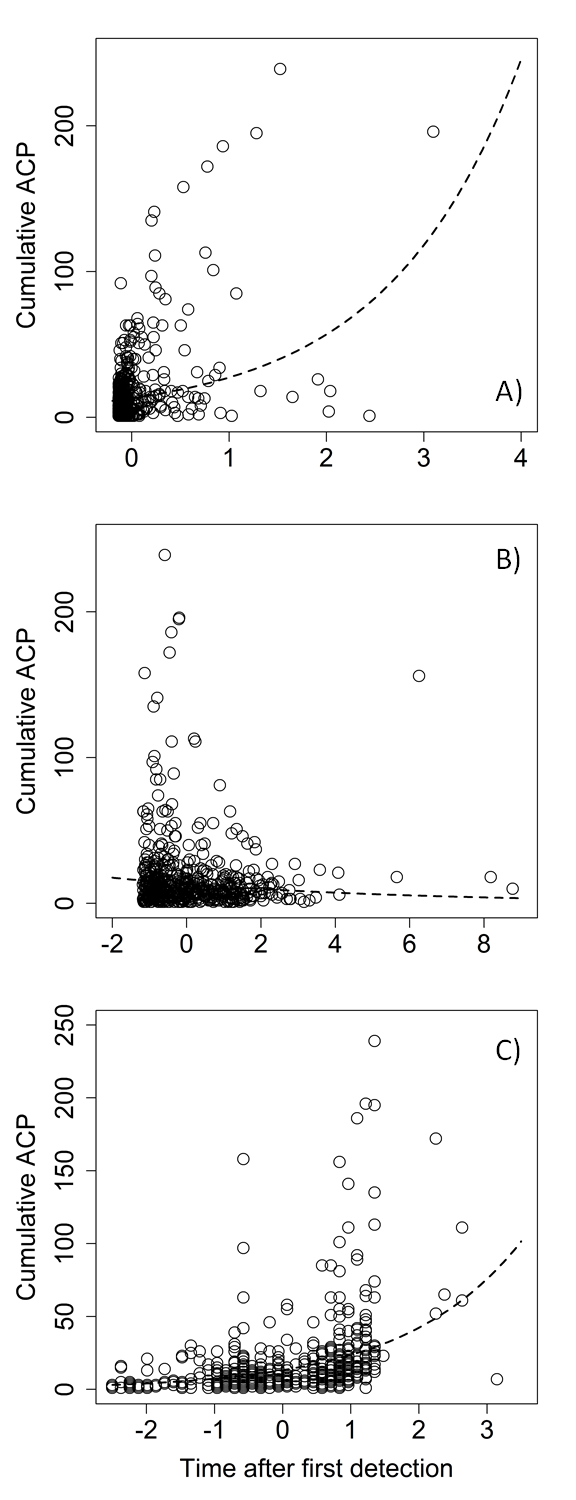

Supplement: Supplementary file 1 [file Data_Sheet_1.docx]
